# Check the diastolic blood pressure twice in aortic dissection as it is associated with prognosis

**DOI:** 10.12669/pjms.37.2.2877

**Published:** 2021

**Authors:** Sezai Sari, Kemal Sener, Mustafa Calis, Mustafa Polat, Adem Kaya, Sadiye Yolcu

**Affiliations:** 1 Sezai Sari, Adana City Research & Education Hospital Deptartment of Emergency Medicine, Adana/Turkey; 2 Kemal Sener, Adana City Research & Education Hospital Deptartment of Emergency Medicine, Adana/Turkey; 3 Mustafa Calis, Adana City Research & Education Hospital Deptartment of Emergency Medicine, Adana/Turkey; 4 Mustafa Polat, Adana City Research & Education Hospital Deptartment of Emergency Medicine, Adana/Turkey; 5 Adem Kaya, Adana City Research & Education Hospital Deptartment of Emergency Medicine, Adana/Turkey; 6 Sadiye Yolcu Adana City Research & Education Hospital Deptartment of Emergency Medicine, Adana/Turkey

**Keywords:** Aortic dissection, diattolic blood pressure, systolic blood pressure, prognosis

## Abstract

**Objective:**

In this study we aimed to determine the prediction level of admission diastolic blood pressure (DBP) on the prognosis and mortality in aortic dissection patients over 65 years old and under 65 years old.

**Methods:**

We included 72 patients in this retrospective study and study groups were divided into two groups according to 65 age. Demographic data, dissection type (Stanford A-B), DBP, systolic blood pressure (SBP), mean arteriel pressure (MAP), heart rate (/min) main complaints, preoperative length of stay, hospitalisation clinic (clinic/intensive care unit), length of hospitaliisation, complications during hospitalisation (renal failure etc..) and the outcome (death/dischargement) results were noted. Preoperative lenth of stay, hospitalisation length, outcome and complications were compared between groups according to SBP, DBP, MAP and heart rate.

**Results:**

Mean blood pressure values of the Stanford type B patients over 65 years old were higher than the other group (p<0.05). Fifty percent of patients under 65 years old were discharged but this ratio was 26.9% in the elder group. DBP was positively correlated with preoperative length of stay and hospitalisation length and negatively correlated with mortality. DBP under 65 mmHg was significantly related with high mortality (p<0.05). When the age and presentation time heart rate is added to each other, the values over 142 were significantly related with high mortality (p<0.05).

**Conclusions:**

The presentation time vital signs especially the DBP may be helpful for emergency clinicians to predict the prognosis and outcome in aortic dissection patients which has high mortality ratio in patients over 65 years of age.

## INTRODUCTION

Aortic dissection (AD), is a cardiovasculary emergency with morbidity and mortality ratios and requires immediately recognition and treatment.^1^ Annually 6000-10000 AD patients have been reported in the US. The annual incidence is 2-3.5/100.000.^2^,^3^ Mortality per hour for the first two days is 1-3%.^4^ It is more common in males and incidence increases with age.^5^ Uncontrolled hypertension is known as the most common risk factor and 65-75% of the patients are hypertensive.^6^ Other risk factors include aortic diseases, aortic valve diseases, aortic family history, cardiovacular surgery history, smoking, pregnancy, connective tissue diseases, direct thorax trauma and iv drug usage.^7^

Hypertension is the most important modifiable risk factor for AD. Antihypertensive therapy reduces the AD risk and is the cornerstone of prevention.^8^ Contraversially, there is limited data about the presentation blood pressure levels and the outcome of the AD patients. On the other hand, presentation time blood pressure may be a useful parameter for the prediction of mortality in these AD patients.^9^

In this study we aimed to determine the predictive value of admission diastolic blood pressure (DAB) on length of hospitalisation, prognosis and outcome in patients under and over 65 years old.

## METHODS

We enrolled AD patients verified via computed tomograpy and hospitalised by cardiovasculary surgery clinic. The exclusion criterias were aortic aneurism, aortic dissection history in past, patients with incomplete data, patients with Marfan syndrome or other connective tissue diseases, bicuspid valve, iatrogenic/traumatic dissections, chronic renal faillure, acute heart failure, acute coronary syndrome. After the Ethics Committee Approval, (Ref: 525, Dated: 28-08-2019) in this retrospective study, we included 72 patients diagnosed with AD between Jan 2019 and December 2019.

### Measurements

The patients data were collceted via hospital data system and patient examination cards. Demographic data (age, gender), ED systolic blood pressure (SBP), diastolic blood pressure (DBP), mean arteriel pressure (MAP), heart rate (HR), classification (Stanford type -B), length of preoperative stay, length of hospitalisation, complication occurence (acute renal failure, stroke, aortic rupture etc..) ratios during hospitalisation and outcome (dischargement/death) results were noted. Blood pressure was measured ambulatory minimally twice from the same arm with the surveillance of a doctor. Patients were divided into two groups according to 65 years old and the Stanford types were determined. Patient groups were compared for DBP, SBP, MAP, HR. length of preoperative stay, length of hospitalisation, complication occurence and outcome accoring to age and Stanford classification. We also aimed to determine a cut-off level for blood pressure that predicts the prognosis and outcome.

SPSS 23.0 (IBM/USA) was used for statistical analyses of the patients’ data. Homogeneity of each parameter was evaluted with Kolmogrow Smirnow and Shapiro Wilks tests. Mean, median, standard deviation were calculated for definitive data. Mann-Whitney-U test was used for quantitative variables and Chi-Square test (X2) was used for qualitative variables. Spearman!s correlation test was used for correlations. Standard methodology was used for improving the clinical decision rule.^10^ First we chose categoric variables those provided p <0.05 in one variable analyses. Then, we chose probable continuous variables with p<0.05 those could be important clinical determinant in one variable analyses. Outcome variables were hospitalisation more than 10 days and mortality. For improving new clinical rules we used measurable parameters and and categoric variables. ROC analyse was used for determining the cut-off level of serum lactate. Sensivity and specivity were calculated. The significance level fort he p was 0.05 with 95% CI.

## RESULTS

In this study, there were 26 (36.1%) female, 46 (63.9%) males with the mean age of 58,13±12,77 (min:19-maks:84). Forty-six (63,9%) patients were under 65 years old and 26 (3.1%) patients were over 65 years old. 84,7% of the patients were Stanford type A, 15.3% were Stanford type B. Eigthyfour point six percent of the over 65 years old group and 84.8% of the under 65 yeras old were Stanford type A.

Mean admission SBP, DBP, MAP and HR of the study group was 124,03±39,52 (min:50-max:230), 73.47±23.80 (minutes:30-max:140), 89,96±28,56 (minutes:36-max:170) mmHg, and 94,11±19,86 (minutes: 43-max:158)/ minutes: respectively. Mean SBP (<65 years old:124.78±36.92, >65 years old: 122.69±44.50), DBP(<65 years old: 74,13±23,43, >65 years old: 72,31±24,86), MAP (<65 years old: 90,67±27,45, >65 years old: 88,69±30,93).

There was no complications in 18 (25%) patients. The complication occurence ratio was 75%. Acute renal failure ratio was significantly higher in the over 65 years old group (p<0.05). Thirty patients (41.7%) were discharged after hospitalisation and 42 (58.3%) patients died intraoperatively or during hospitalisation.

Length of preoperative stay was not significantly differen between age groups (p>0.05). Twenty-three (50%) of the under 65 years old group and 7 (26.9%) of the over 65 years old group were discharged and this difference was significantly different between groups (p<0.05). Similarly, 23 (50%) of the under 65 years old group and 5 (19.2%) of the over 65 years old group died and the difference was significantly different.

When we evaluated the patients according to Stanford type SBP, DBP and MAP values were significantly lower in Stanford A dissections (p<0.05). Mean values of SBP, DBP, MAP, HR, hospitalisation day, length of preoperative stay and complication occurence status of the patients according to Stanford classification is given in Table-I.

**Table-I:** Comparison of SBP, DBP, MAP, HR , hospitalisation day, length of preoperative stay, complication and the outcome of the patients according to Stanford classification.

	*Stanford A Mean (min-max)*	*Stanford B Mean (min-max)*	*P*
SBP (mmHg)	119,01	151,82	0,012*
	(50-230)	(100-180)	
DBP (mmHg)	70,49	90	0,014*
	(30-140)	(70-110)	
MAP (mmHg)	86,31	110,18	0,015*
	(36-170)	(80-133)	
HR (at1m/dk)	94,90	89,73	0,656
	(43-158)	(75-101)	
Length of hospitalisation	11,47	13,64	0,409
	(1-85)	(1-40)	

	*Stanford A >n (%)*	*Stanford B n (%)*	

***Preop length of stay***			
0-24 h	30 (%49,18)	1 (%9,1)	0,008*
24-48 h	23 (%37,70)	4 (%36,4)	
48-72 h	1 (%1,64)	1 (%9,1)	
No operation	7 (%11,48)	5 (%45,4)	
***Complication***			
Yes	45 (%73,77)	8 (%72,73)	0,942
No	16 (%26,23)	3 (%27,27)	
***Outcome***			
Discharged	25 (%40,98)	5 (%45,45)	0,782
Eksitus	36 (%59,02)	6 (%54,55)	

*: p<0,05. preop: preoperative, SBP:systolic blood pressure,

DBP:diastolic blood pressure, MAP: mean arteriel pressure

The length of preoperative stay was significantly between Stanford A and B in both age groups (p<0.05). Seventeen Stanford A of under 65 years old patients group were operated between 24-48 hours of presentation (Table-II).

**Table-II:** The length of preoperative stay between Stanford A and B according to age groups

			*Preoperative time*				*Total*	*P*

			*0-24h*	*24-48h*	*48-72h*	*No Op.*	
<65 Years	Stanford	A	18	17	1	3	39	<0,05
		B	1	3	0	3	7	
	Total		19	20	1	6	46	
≥65 Years	Stanford	A	12	6	0	4	22	<0,05
		B	0	1	1	2	4	
	Total		12	7	1	6	26	
Total	Stanford	A	30	23	1	7	61	<0,05
		B	1	4	1	5	11	
	Total		31	27	2	12	72	

We couldn’t find a difference between age groups for the length of hospitalisation, complication occurence and outcome according to Stanford classification (p>0.05). Mean SBP (140,00±31.94 vs 112,62±40.78), DBP (82.00±67.38 vs 67.38±24.50), and MAP (100.93±23.48 vs 82.12±29.52) were higher in discharged patients when compared with exitus patients (p<0.05). Mean HR (86.97±13.60 vs 99.21±22.10) was lower the discharged patients (p<0.05).

There was no significance in mean SBP, DBP, MAP and HR of the patients according to occurence of complication (p>0.05). Mean SBP, DBP, MAP and HR values were significantly different in patients hospitalised more than ten days than patients hospitalised less than 10 days (p<0.05). (Table-III).

**Table-III:** Comparison of SBP, DBP, MAP and HR According to Hospitalisation Length.

	*Length of Hospitalisation (day)*				

	*≥10 days (n=31)*		*<10 days (n=41)*		*P*

	*Mean*	*Sth. Dev.*	*Mean*	*Sth.dev.*	
SBP	139,03	36,273	112,68	38,472	0,004*
DBP	81,29	22,471	67,56	23,322	0,014*
MAP	100,13	26,529	82,27	27,915	0,007*
HR	86,68	14,570	99,73	21,586	0,003*

Positive and negative correlations between the parameters (blood pressure, heart rate, mortality, preoperative time, length of hospitalisation) is given in Table-IV.

**Table-IV:** Correlations between Main Parameters.

	*Preoperative time*	*Complication*	*Mortality*	*Length of hosp. (day)*
Age	(+) 0,805	(+) 0,136	(+) 0,082	(-) 0,644
SBP	(+) 0,001**	(-) 0,131	(-) 0,002**	(+) 0,005**
DBP	(+) 0,001**	(-) 0,127	(-) 0,004**	(+) 0,010 *
MAP	(+) 0,000**	(-) 0,123	(-) 0,003**	(+) 0,008**
HR	(-) 0,011*	(+) 0,141	(+) 0,003**	(-) 0,007**

**Figure 1:**
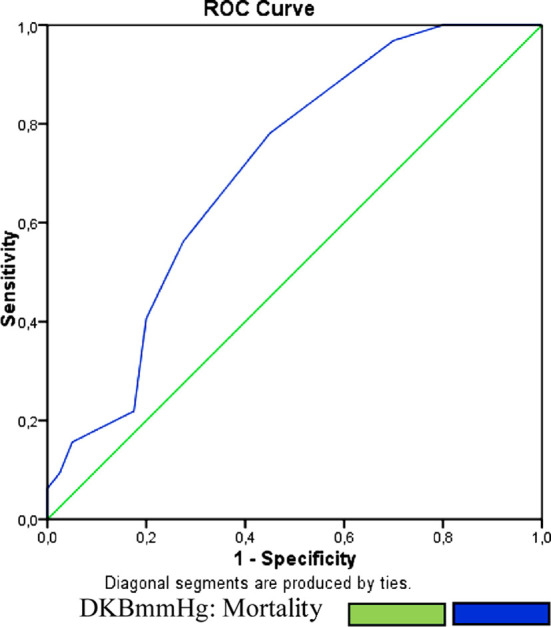
ROC Curve of DBP for mortality.

According to the ROC analyse, DBP <65mmHg is 80% sensitive and 55% spesific for the prediction of mortality in the AD (Fig.1). In the AD patients age+HR formula is related with mortality. Cut-off value fort his formula is 142 in the ROC analyse. Sensivity 58% and the specivity is 81% for the mortality prediction in the AD patients (Fig.2).

**Figure-II:**
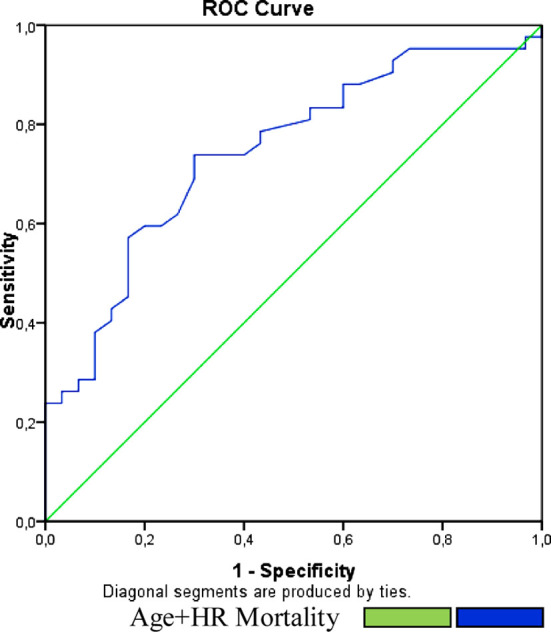
cROC Curve of Age+HR fort he prediction of mortality in the AD.

## DISCUSSION

AD’s mortality rate is 8% for first hours, 13% for first 12 hours, 21% for first 24 hours and 74% for two weeks if not treated immidiately.^4^,^11^ Mortality of type A AD is around 25%. Rajendra reported 32.5% of postoperative mortality ratio.^12^ Our single-center study’s mortality ratio was 58.3% because we included intraoperative deaths. Mortality is higher in elderly patients more than 70 years old when compared with younger age groups.

Hirst et al, reported that mortality increases over 70 years old AD patients.^13^ In another study borderline age as given as 65 for higher risk for mortality.^14^ Our study mainly compared patients under and over 65 years old. And the mortality of the patients over 65 years old was higher than the orther group. These elderly patients have several comorbidities, unsufficient hemodinamic compansation. Old age is also associated with morbidity. Renal failure incidence is 10% for type B and 20% for A AD.^15^ This renal failure maybe the result of hypoperfusion, infarction, prolonged hypotension or renal artery dissection So, urinary output and serum creatinin levels preand postoperatively in AD patients carefully. Hypertension is the most important risk factor for AD. SBP should be maintained under 120mmHg and the HR under 60. Hypertension accompanies Stanford B much more than tye A. On the other hand 20% patients present with hypotension.^16^ The reasons of low blood pressure are aortic rupture, myocardial ischemia, aortic insufficience, and pericardial tamponade. According to Bossone et at’s study inhospital mortality and complication ratio is higher in hypotensive patients.^17^ In another study patients over 65 years old whose SBP is ≤80 mmHg are under higher risk for inhospital mortality.^14^ Our study suggests similar results for hypotensive AD but we coulnd’t find a relation between hypotension and complication occurence.

High HR in the AD increases the aortic wall distress so a HR lower than 60/minutes is suggested.^18^ In our study, similar to literatüre, high HR is related with mortality. Type A AD patients over and under 70 years didn’t differ according to hospitalisation length. The authors enrolled 464 patients in 2011.^19^ According to these data, it can be said that hypotensive tachycardic AD patients’ prognosis are worse than hemodinamically stable patients. The mortality and hospitalisation length risk is arised.

Recently, the effect of low DBP on morbidity and mortality in acute cardioovasculary diseases.^19^-^22^ Low DBP is associated with high mortality and longer hospitalisation length and it is one of published 21 predictive values.^23^ Similarly, according to a 2017 study which included 6177 patients, low DBP is closely related with subclinical myocardial ischemia.^22^ Low blood pressure may be a sign for t he thickness of the aortic wall which is an important risk factor for dissection may trigger it. Lu et al reported that, low DBP is associated with higher 3rd, 24th and 60th month complications.^24^ They enrolled 269 type B AD patients and according to their study, DBP at admission is correlated with aortic-related adverse events after thoracic endovascular aortic repair. In our study cut-off value of DBP was 65 mmHg for predicting the mortality with 80% sensivity and 55% specivity.

We also reported a bordeline for age+HR. Patients whose age+HR more than 142 are under high risk for higher mortality with 81% sensivity and 58% specivity. This value may easily calculated fort he AD patients in the ED.

### Limitations of the Study

In this retrospective single center study, most of our AD patients were Stanford Type-A, so the homogeneity wasn’t provided enough.

## CONCLUSION

DBP may be helpful for emergency clinicians to predict the mortality in AD patients which has high mortality ratio in patients over 65 years old. Admission low blood pressure values suggested high mortality, the initial assessment and resuscitation of these patients should be made much more carefully. Further comprehensive prospective studies are warranted for risk assessment in AD patients.

### Author Contribution:

**SS, KS, SY:** Conceptualization, Methodology, takes the responsibility for integrity of the study.

**SS, MC, MP:** Data Curation.

**SS, KS, MC, AK:** Statistical Analyse.

**SY, AK:** Writing-editing.

All authors declare that they have no conflict of interest.
